# Study on the Imprinting Status of Insulin-Like Growth Factor II (IGF-II) Gene in Villus during 6–10 Gestational Weeks

**DOI:** 10.1155/2010/965905

**Published:** 2010-07-08

**Authors:** Jianhong Chen, Qun Fang, Baojiang Chen, Yi Zhou, Yanmin Luo

**Affiliations:** Department of Obstetrics and Gynecology, Fetal Medical Center, First Affiliated Hospital, Sun Yat-sen University, Guangzhou 510275, China

## Abstract

*Objective*. To compare the difference of imprinting status of insulin-like growth factor II (IGF-II) gene in villus between normal embryo development group and abnormal embryo development group and to investigate the relationship between karyotype and the imprinting status of IGF-II gene. *Methods*. A total of 85 pregnant women with singleton pregnancy were divided into two groups: one with abnormal embryo development (*n* = 38) and the other with normal embryo development (*n* = 47). Apa I polymorphism of IGF-II gene in chorionic villus was assayed with reverse transcriptase polymerase chain reaction (RT-PCR) and restriction fragment length polymorphism (RFLP). The relationship between chromosomal abnormal karyotype and IGF-II gene imprinting status was analyzed by primary cell culture and G-banding chromosomal karyotype analysis. *Results*. IGF-II imprinting loss rate was higher in the abnormal embryo development group than the normal embryo development group (44.7% versus 31.6%), but without significant difference (*P* > .05). The percentage of abnormal chromosomes of chorionic villus in the abnormal embryo development group was 42.5%, in which IGF-II imprinting loss rate reached 64.7%. No abnormal karyotypes were found in the normal embryo development group. However, there was significant difference in IGF-II imprinting loss rate between two groups (*P* > .05). *Conclusion*. During weeks 6–10 of gestation, abnormal embryonic development is correlated with chromosomal abnormalities. The imprinting status of IGF-II gene played important roles in embryonic development, and imprinting loss might be related to chromosomal abnormalities.

## 1. Introduction

Genomic imprinting (also, namely, gene imprinting) is an epigenetic phenomenon inconsistent with the traditional Mendelian inheritance. The definition of genomic imprinting is that alleles from father or mother are modified when they transmit genetic information to offspring, resulting in only one of alleles from father or mother expressed in offspring [1]. Imprinting is a dynamic process. It must be possible to erase and re-establish the imprint through each generation. The nature of the imprint must therefore be epigenetic (modifications to the structure of the DNA rather than the sequence). The majority of imprinted genes are related with embryonic development. The parental-specific expression is obtained through epigenetic modifications (DNA methylation, histone tail modifications) which alter the conformation of chromatin fiber and therefore regulate the expression of the underlying genes. Deletions, duplication, mutations, or alterations of imprinting of the only active allele as well as uniparental sisomy or loss of imprinting of the inactive allele lead to and unbalance (loss of function or gain of function) in the dosage of the gene product and may have phenotypic consequences.

Insulin-like growth factors (IGFs) are polypeptides that play an important role in cellular proliferation and differentiation. Insulin-like growth factor II (IGF-II) is an important factor of human early embryo and placenta development, and its roles are closely related with the gene imprinting status [2]. Thus, gene imprinting loss will lead to abnormal embryonic development. During the process of human fetal development, 3 to 8 gestational weeks are the most important. Any factor impacting nutrition supply and transmission of embryonic growth and development can result in abnormal differentiation of embryos and abortion. Spontaneous abortion accounts for 10–15% of all pregnancies and the majority of them are early spontaneous abortion. At present, embryonic chromosomal abnormality is a recognized etiological factor. Therefore, we detected IGF-II gene imprinting status and chromosomal karyotype of villus in patients with embryonic growth arrest and investigate the relationship between them.

## 2. Materials and Methods

### 2.1. Subjects

Case group: 47 pregnant women who visited outpatient clinic of family planning and eugenic genetics in First Affiliated Hospital of Sun Yat-sen University because of single gestation sac, empty embryo pregnancy, and embryonic development arrest from April 2002 to January 2004 were enrolled in this study. The informed consent was obtained from all subjects before participating in this study. The inclusion criteria of this group included: B-type ultrasonography that revealed embryonic growth arrest or no embryo observed by repeated B-type ultrasonography; regular menstrual cycle 3 months before pregnancy; 6–10 gestational weeks; no case history of endocrine diseases and cancer; no administration with hormone drugs during pregnancy; serum Toxplasma (TOX), Nubbavirs (RV), Cytomegalo virus (CMV), Herpes simplex virus (HSV) infection detection was negative. In this group, the mean age was 26.7 + 0.2 years and the mean gestational age was 7.2 + 0.1 weeks. 

Control group: at the same time, 38 women of normal early pregnancy with single gestation sac were randomly selected. B-type ultrasonography revealed that embryonic development was normal as well as embryo and fetal heart beat. In this group, the mean age was 27.3 + 0.3 years and the mean gestational age was 6.9 + 0.2 weeks. There were no significant differences in age, gestational age, and number of pregnancies between the two groups.

### 2.2. Methods

#### 2.2.1. Chorionic Villus Cell Culture and Chromosome Analysis

 Chorionic villus cell culture: During the termination by artificial abortion, chorionic villi were collected under sterile conditions then dissected under a dissecting microscope. The dissected chorionic villi were separated and washed repeatedly with ice-cold normal saline to remove attached blood and deciduas, then shredded, digested with 0.25% trypsin for 5 min, followed by centrifugation at 1000 r/min for 10 min, and discarding supernatant. Subsequently, 1 ml collagenase II (sigma company, USA) was added into the sediments. 5 min later, supernatant was removed after centrifugation at 1000 r/min, and the sediments were resuspended in 2 ml complete medium (Complete Amnio Max, Invitrigen company, USA), and cell suspension was transferred into two flasks (about 1 ml). Cell growth was daily observed under inverted microscope. When cell fusion and good vitality were found in 40%–50% of cells, the cells were harvested for chromosome analysis.

G-banding chromosome preparation: Colchicine (0.15 *μ*g/ml final concentration) was added in the harvested cell suspensions. Subsequently, the mixture was put in a 37°C water bath for 60 min, followed by adding 2 ml prefixing liquid, mixing, centrifugation, discarding supernatant, fixation with 6 ml 1 : 3 fixing solution for 2 min, centrifugation for 10 min, discarding supernatant, adding 60% acetic acid, 2 ml methanol 2-3 min later, mixing, centrifugation, discarding supernatant, fixation with 4 ml 1 : 3 fixing solution for 1 h, discarding supernatant, preparing 1-2 conventional G-banding chromosome. At least 15 splitphases were observed, and karyotype analysis was performed in 3 phases in each specimen.

#### 2.2.2. Detection of IGF-II Gene Polymorphism

 Chorionic villus genomic DNA was extracted with saturated phenol and chloroform, and then IGF-II gene polymorphism was detected by polymerase chain reaction (PCR)-restriction fragment length polymorphism analysis. The total volume of PCR reaction system was 50 *μ*L, including template DNA 600 ng, 10× PCR reaction solution 5 *μ*L, 10 U/*μ*L Taq enzyme (TaKaRa Inc., Japan) 0.5 L, 10 mmol/L dNTPs 1 *μ*L, upstream primer 1 *μ*L, and downstream primer 1 *μ*L (design for Apa I polymorphic restriction site of 9th exon of IGF-II gene). PCR reaction conditions were as follows: predenaturation at 94°C for 5 min, 35 cycles of denaturation at 94°C for s, annealing at 55°C for 40 s and extension at 72°C for 40 s, final extension at 72°C for 7 min. IGF-II gene PCR products (236 bp) were digested overnight at 25°C with restriction enzyme ApaI (BioLabs). Subsequently, their genotypes were analyzed with 1.5% agarose gel electrophoresis. IGF-II genes expressed diallele (236 bp, 173 bp and 63 bp fragments (type AB)), indicating that the loss of imprinting occurred while monoalle (only 236 bp fragment (type A) or only 173 bp and 63 bp fragments (type B)), indicating normal imprinting status. IGF-II upstream and downstream primers were 5′-CTTGGACTTTGACTCAAATTGG-3′and 5′- CCTCCTTTG GTCTTA CTG GG -3′, respectively.

#### 2.2.3. IGF-II Gene Imprinting Status Detection

**Figure 1:**
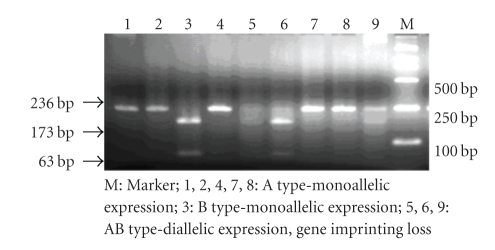
IGF-II gene imprinting status detected by RT-PCR.

Chorionic villus IGF-II gene imprinting status was detected by reverse transcription-polymerase chain reaction (RT-PCR) and ApaI restriction enzyme digestion. Heterozygous genomic DNA specimens (type AB) were selected to extract RNA. QIAGEN Poly A^+^ mRNA extraction kit (Germany) and RT-PCR (two-step) kit (Invitrigen company, USA) were used in this study following the instructions. PCR reaction and Apa I restriction enzyme digestion method of RT-PCR products were similar to IGF-II gene polymorphism detection (Figure 1).

#### 2.2.4. Statistical Analyses

All statistical analyses were performed by SPSS version 13.0 statistical software. The data in each group is presented as the mean ± SD, and comparison between groups was determined by *χ* ^2^ test, with *P* < .05 considered significant.

## 3. Results

### 3.1. Primary Culture of Chorionic Villus Cells and Chromosome Karyotype Analysis

 In this study, a total of 47 cases of chorionic villus were cultured in the abnormal embryonic development group, including culture failure in 6 cases, primary culture failure in 1 case because of villus degeneration, with a failure rate of 15%, while 38 cases in the normal embryonic development group were successfully cultured. The mean culture time in abnormal and normal embryonic development groups was 13.1 ± 1.4 d and 6.8 ± 0.9 d, respectively. Obviously, the former was twice longer than the latter in culture time, which might be related with the long time embryonic death, would cause villus necrosis, low activity. 

G-banding chromosome karyotype analysis of chorionic villus was normal in the normal embryonic development group while abnormal karyotype was 17 cases (42.5%) in the abnormal embryonic development group, including male karyotype in 6 cases, female karyotype in 5 cases, 45, XO in one case, 69, XXX in three cases and 69, XXY in 2 cases. One case of cat cry syndrome suffered from dead fetus at one time and induced abortion was performed in second pregnancy because cord blood chromosome analysis revealed cat cry syndrome. This unwanted pregnancy expressed embryonic development arrest, and its chorionic villus chromosomes were 46, XX and del (5) (p15). The detailed karyotypes were as shown in Table 1.

### 3.2. Villus IGF-II Gene Imprinting Status

 In the abnormal embryonic development group, the imprinting loss rate of IGF-II gene was 44.7%, which was higher than 31.6% in the normal group, but without significant difference (*χ* ^2^ = 1.52, *P* > .05). Gene imprinting loss rate of embryonic villus karyotype was similar between male and female, without significant difference (Table 2).

The chromosomal abnormality rate was 42.5% in the abnormal embryonic development group. Of 17 specimens with abnormal karyotypes, 11 cases suffered from IGF-II gene imprinting loss, with the loss rate up to 64.7%. While, no abnormal villus chromosomal karyotype analysis was found in the normal embryonic development group. There was no significant difference between two groups (*χ* ^2^ = 5.3, *P* < .05).

## 4. Comment

### 4.1. Primary Culture of Chorionic Villus Cells in Embryonic Development Arrest

The culture time of chorionic villus cells from abortion or dead fetus depended on cell survival rate. The culture time was up to 3 to 4 weeks if there were less active cells in tissues. Culture failure was defined as cell growth arrest for more than one month [3]. Therefore, timely specimen collection was crucial for successful chromosomal detection for villus in embryonic development arrest. In 2002, Greenwold and Jauniaux [4] reported that ultrasound-guided sucking villus from early spontaneous abortion could obtain only 4.5% of villus cell culture failure rate. In this study, the specimens were collected by vacuum uterine aspiration, which was easily polluted by vaginal secretions. However, intrauterine collection for villus could reduce the risk of pollution and improve culture survival rate of villus.

 Villus culture in 40 cases of embryonic development arrest showed that chromosomal abnormality was 42.5%, indicating that nearly one half of fetus with chromosomal diseases (the majority was severe chromosomal abnormalities, resulting in severe imbalance of genetic materials) could not survive to birth, due to natural selection. Except chromosome 1, triplont was found in all human chromosomes. Of them, triplont in chromosome 1 accounted for one third of all triplonts and was one kind of highly lethal triplont. The other common triplonts included triplonts in chromosomes 21 and 22 [5]. Except triplont in chromosome 21, other triplonts could not survive [6]. Autosomal triplont was the most common chromosomal abnormality in embryos. The experimental findings in this study confirmed this phenomenon.

### 4.2. IGF-II Gene Imprinting Loss and Embryonic Development

The balance of cell apoptosis and proliferation was crucial to maintain pregnancy, and its functional regulation disorder might lead to early embryonic development arrest. Warner deemed that the dynamic balance of speed of development and apoptosis was an internal factor of embryo survival. In the process of embryonic development, development and apoptosis-related genes, cytokines and corresponding receptors played important roles in embryonic survival [7]. 

Imprinted genes of germ cells cycled with reproductive cycle through three phases of erase, formation, and maintenance. After fertilization, the majority of genes experienced re-demethylation, while imprinted genes maintained original methylation status before and after fertilization and nidation due to the protection of differentially methylated regions. Meanwhile, internal and external environment changes could lead to genetic alternations and gene imprinting loss. This study showed that IGF-II gene imprinting loss in the embryonic development arrest group was slightly higher than the normal group, without significant difference. However, IGF-II gene imprinting loss of 17 abnormal karyotypes in the abnormal embryonic development group was significantly higher than the normal embryonic development group. Thus, we speculated that chromosomal abnormalities of embryos could result in abnormal function expression of regulatory genes in early embryonic development, leading to increased IGF-II gene imprinting loss, which might destroy the balance between villus and deciduas, leading to shallow embryo implantation, spontaneous abortion, and embryonic development arrest. However, the collected data in this study was relatively less, and thus there should be a bigger sample size, to further discuss and explore whether IGF-2 imprinting loss was a new molecular genetic marker of spontaneous abortion or not. 

Some scholars believe that IGF-II gene imprinting loss leads to overexpression [8, 9] because gene imprinting loss activates the original silent mother-derived alleles, resulting in two times of gene expression level and significantly elevated IGF-II protein expression. It was also deemed that IGF-II gene imprinting loss could result in overgrow diseases, including Beckwith-Wiedemann syndrome (BWS) as well as “fetal overgrowth syndrome”, resulting in prenatal overgrowth, polyhydramnios, fetal, and neonatal deaths. However, some scholars also concluded that there were no significant correlation between IGF-II gene imprinting loss and IGF-II protein overexpression, and even IGF-II gene imprinting loss resulted in the downregulation of IGF-II protein expression [10–12]. Vambergue A et al. found that no loss of genomic imprinting of IGF-II in placentas of diabetic pregnancies with fetal macrosomia [13].

There might be a lot of complicated middle links between IGF-II gene imprinting loss and IGF-II expression regulation.

## Figures and Tables

**Table 1:** Villus karyotype analysis of embryonic maldevelopment.

Chromosomal abnormality type	Caryotype	*n*
Trisomy	47, XX (XY), +21	4
	47, XX (XY), +8	1
	47, XX (XY), +13	2
Triplont	69, XXX	3
	69, XXY	2
Haplotype	45, XO	1
Other	46, XY, t (9; 22) (p13; p12)	1
	46, XX/46, XY	1
	46, XX, del (5) (p15)	1
	46, XX, i(10)(qter→cen→qter)	1
Total		17

**Table 2:** IGF-II gene imprinting status in the abnormal and normal embryonic development groups.

Group	Genotype AA/BB		AB	
	*n*	%	*n*	%
Normal group	26	68.4	12	31.6
Abnormal group	26	55.3	21	44.7
(chromosomal abnormality)	6	35.3	11	64.7

A, B, and AB indicates three kinds of genetic imprinting status, AA/BB indicate normal imprinting, AB indicates gene imprinting loss.
